# Correction: Inhibition of mTOR Alleviates Early Brain Injury After Subarachnoid Hemorrhage Via Relieving Excessive Mitochondrial Fission

**DOI:** 10.1007/s10571-024-01477-2

**Published:** 2024-04-22

**Authors:** Yuchen Li, Pei Wu, Jiaxing Dai, Tongyu Zhang, Ji Bihl, Chunlei Wang, Yao Liu, Huaizhang Shi

**Affiliations:** 1 Department of Neurosurgery, The First Affiliated Hospital of Harbin Medical University, Harbin, 150000 Heilongjiang China; 2 Department of Pharmacology and Toxicology, Boonshoft School of Medicine, Wright State University, Dayton, OH 45435 USA

**Correction to: Cellular and Molecular Neurobiology (2020) 40:629–642** 10.1007/s10571-019-00760-x

The original version of this article unfortunately contained error in Fig. 4.

In Fig. 4a, the pictures of DAPI in Sham and SAH + rapamycin were repetitive, but the pictures of TUNEL and Merge were correct.

The corrected Fig. 4 is presented here with the correct image of DAPI in SAH + rapamycin and this error did not influence the experimental results and conclusions.

**Fig. 4:**
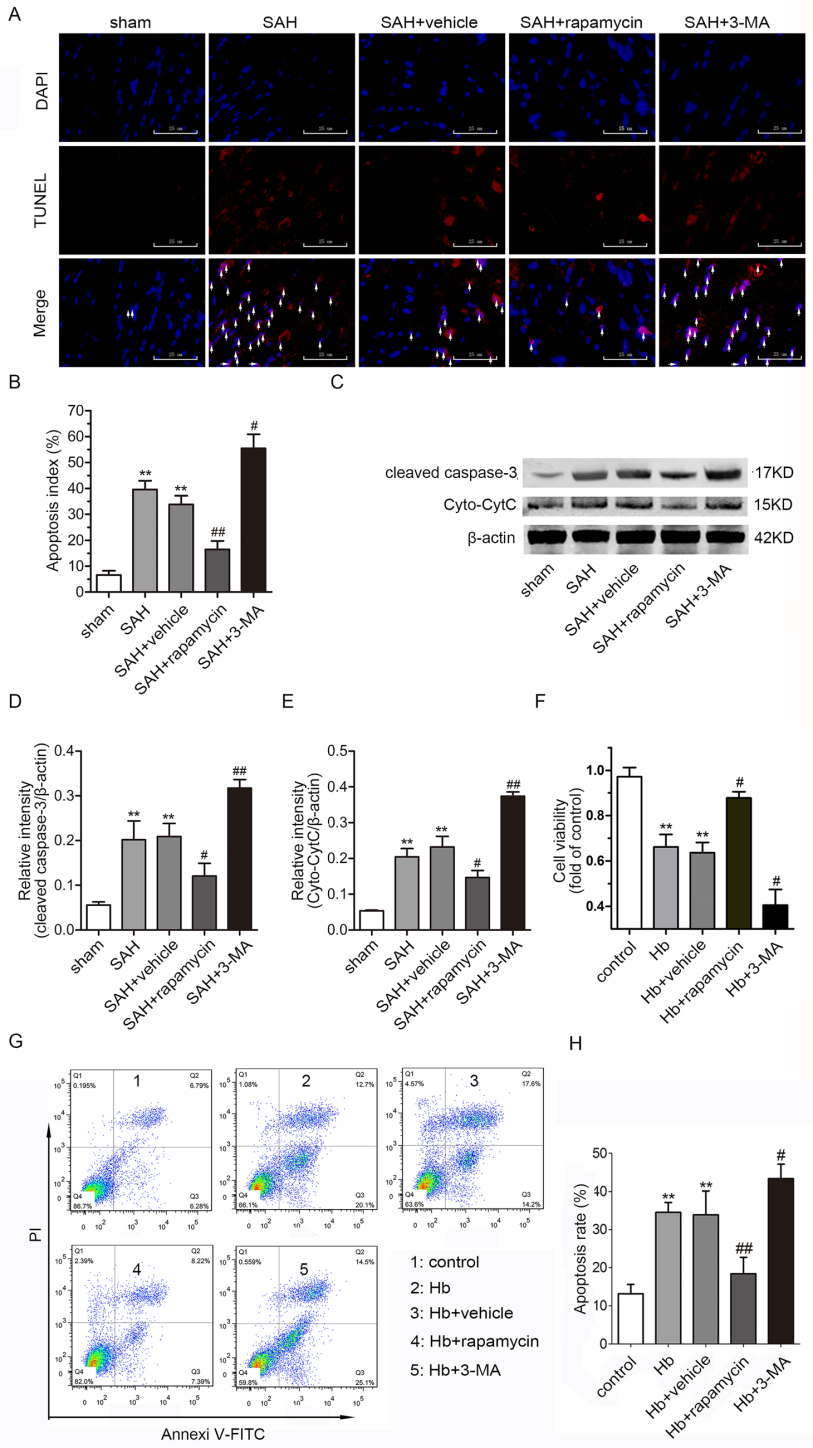
Inhibition of mTOR-alleviated neuronal apoptosis after SAH. **a** Representative photographs of TUNEL staining in brain sections (scale bar = 25 μm). **b** Apoptosis index of the TUNEL staining in each group (*n* = 3). **c** Representative images of Cyt C and cleaved caspase-3 bands in rat neurons. **d** Quantification of the cleaved caspase-3 expression (*n* = 6). **e** Quantification of the Cyt C expression (*n* = 6). The results were normalized to β-actin. **F** The cell viability in each group was detected by the MTT assay (*n* = 6). **g** The apoptotic rate was assessed by flow cytometry. **h** Statistical analysis of the apoptotic rate in each group (*n* = 6, **p < 0.01 vs. sham/control, ^#^*p* < 0.05 and ^##^*p* < 0.01 vs. SAH/Hb)

The original article has been corrected.

